# *Afro Algorithms*: Imagining new possibilities for race, technology, and the future through animated storytelling

**DOI:** 10.1016/j.patter.2021.100327

**Published:** 2021-08-13

**Authors:** Anatola Araba Pabst

**Affiliations:** 1 Visual Storyteller, New York, NY, USA

## Abstract

Enter the world of *Afro Algorithms*, a 3D-animated short film created by multimedia artist and filmmaker Anatola Araba Pabst. The film explores Afrocentric visions for the future while advocating for a new standard of leadership, the importance of diverse perspectives, how creativity is our greatest superpower, and more. Embark on a journey that at once entertains, enlightens, inspires, and educates.

## Main text

The year is 2220. Robots serve as crossing guards and humans jaunt on hoverboards through the city streets. Tomorrow, people from far and wide will tune in to a very special broadcast: the inauguration speech of Aero, the world’s first AI leader.

Welcome to the world of *Afro Algorithms*. This 3D-animated short film in the Afrofuturist genre explores the topics of AI and bias. Through an enchanting science-fiction aesthetic, this story follows the journey of Aero, the first artificial intelligence leader of Earth, as she discovers who she really is—beyond her programming.

Commonly referred to as the lovechild between *Black Panther* and *I, Robot*, this short film explores Afrocentric visions for the future while advocating for a new standard of leadership, the importance of diverse perspectives, how creativity is our greatest superpower, and much more.

My name is Anatola. I am a multimedia artist, filmmaker, and futurist from New York City. I am also the creator of *Afro Algorithms*. While in my senior year at NYU, I went into a deep dive researching the pros, cons, and controversies of artificial intelligence. I sourced inspiration from nuanced studies on race, technology, and the social implications of AI. The most influential of these works are provided at the end of this article for further exploration on this topic. I distilled the insights from these studies into *Afro Algorithms*, with the aim of creating a work of science fiction that at once entertains, enlightens, inspires, and educates.

AI is powered by data, and data are a reflection of the past. For some matters, like being able to tell the difference between a rose and a tangerine, this is totally fine. The AI-powered algorithm is simply taught the history of how roses and tangerines look respectively and eventually can distinguish between the two. But for other matters, like what resume should be considered for a job interview or who may be a suspect for a crime, things aren’t quite as simple.

Earth is a planet that has been plagued with inequalities for millennia. Inequalities across demographics, “us” versus “them,” race, ethnicity, religion, gender, and class. Historical data illustrate to AI that some level of discrimination is, well, just how things are done around here. Therefore, AI will dutifully continue to perpetuate oppressive behavior toward certain groups into the future unless the technology, or human nature, transforms for the better.

In the world of *Afro Algorithms*, Aero is voiced by Ava Rain, a musician with a record-breaking album and former background vocalist to Solange, Carly Rae Jepson, Blood Orange, among others. Aero is programmed with the data of every great leader throughout history, or so she has been led to believe. Aero’s lead scientist and mother-figure, Miriam, is voiced by long-standing radio personality on *The Howard Stern Show*, Robin Quivers. Miriam is confident that she has programmed Aero with all the knowledge she needs to be a better ruler than anyone who has come before her.

However, the day before her inauguration, Aero realizes that there is important information missing from her databank. Discovering this, Aero embarks on a mission to find her missing data and complete her algorithm before her inauguration speech. This journey leads her to a scientist named Dr. Richards, voiced by Angolan actor and African Oscar-winner Hoji Fortuna. Dr. Richards shows Aero that a whole range of pivotal perspectives are missing from her algorithm. So long as she remains the same, she will be unable to create real change.

Dr. Richards reveals the most important part of Aero’s programming: the codes of creativity, imagination, and vision that lay dormant within her. By activating her creativity, she will no longer be limited to the data of the past while building the future. Only by utilizing the power of creativity can Aero bring forth a future the likes of which has never been seen before.

While human beings are far from AI, humans and machines do have quite a lot in common. Humans interpret vibrating waveforms through the senses to create what we call reality. Just as the programming of a computer dictates the way it performs, our reality is largely determined by our sociocultural programming.

However, by thinking critically on a personal and societal level about the things we do and why we do them, we will find that numerous practices of life and society ought to be transformed, streamlined, or even removed altogether. It is not easy to change, but it can be done by embracing a spirit of innovation and courage.

In the United States, Black people have historically been subjected to the dark side of society’s programming. In turn, the highly melanated among us are especially vulnerable to the increasing power of AI technology. In some cases, the problem is that there is a lack of diverse data, which causes AI to discriminate. In other cases, the problem is that Black people appear abundantly within datasets that don’t exactly portray people with darker complexions in the best light.

For instance, some of the world’s most powerful and widely used facial-recognition technology is partially blind when it comes to recognizing Black faces.^1^ This is because the people who programmed the world’s leading facial recognition technology failed to provide adequate representation of Black faces when teaching the AI how a face is supposed to look. On the other hand, AI used by police departments to identify criminals endangers Black people in particular of being accused of crimes they did not commit. This is because these AI learn what a criminal looks like from prison data, and if you know anything about prisons in the US, you know that they disproportionately contain Black and Brown bodies.^2^ This leads to horrific cases like that of Robert Julian-Borchak Williams, who was arrested in Michigan in front of his wife and kids for a crime he never committed, all due to the fault of an algorithm.^3^

In this regard, building more ethical AI is not only about having more or less representation of Black data. Rather, it is about *how* these machines are programmed to use the data they are given.

This brings us back to the core message of *Afro Algorithms*: we should no longer rely on the data of the past to build the future. Let us have the courage to live, behave, and build things to be more ethical, sustainable, and inclusive than they have ever been before. Let us reimagine the world as it has the potential to be.

AI is not going anywhere any time soon. It has become our assistant when writing emails, our wingman when swiping through dating apps, and hauntingly, an overseer that can identify you in a crowd through a camera far in the distance. As AI plays an increasingly larger role in our society, it is pivotal that we are aware of the impact it can have on us and on future generations.

Whether human beings or machines, we can all learn something from Aero’s journey in *Afro Algorithms*. After all, I created this story with the intention of sparking important conversations about race, technology, and where humanity is driving the future of this planet.

*Afro Algorithms* will be screening at select film festivals before it is available publicly online. To catch an exclusive screening or to be notified when the film is released, sign up for updates at https://www.afroalgorithms.com/ or follow the project on Instagram at @afroalgorithms. If you have a question or inquiry, please email afro.algorithms@gmail.com.

For further exploration on this topic, see the following resources:

Book: *Race After Technology*, written by Ruja Benjamin
Book: *Algorithms of Oppression*, written by Safiya Noble
Book: *Artificial Unintelligence*, written by Meredith Brussard
TED Talk: “How I’m Fighting Bias in Algorithms,” spoken by Joy Bolumnwini
Documentary: *Coded Bias*, directed by Shalini Kantayya

## References

[1] MIT Media Lab Results. https://www.media.mit.edu/projects/gender-shades/results/.

[2] Pariona A. (2019). US Prison Population By Race. https://www.worldatlas.com/articles/incarceration-rates-by-race-ethnicity-and-gender-in-the-u-s.html.

[3] Hill K. (2020). Wrongfully Accused by an Algorithm. https://www.nytimes.com/2020/06/24/technology/facial-recognition-arrest.html.

